# Ultra-Wideband Power Amplifier Design Strategy for 5G Sub-6-GHz Applications

**DOI:** 10.3390/mi13091541

**Published:** 2022-09-17

**Authors:** Jorge Julián Moreno Rubio, Edison Ferney Angarita Malaver, Jairo Alonso Mesa Lara

**Affiliations:** Grupo de Investigación en Telecomunicaciones—GINTEL, Universidad Pedagógica y Tecnológica de Colombia, Sogamoso 152211, Colombia

**Keywords:** GaN-based FETs, ultrawideband power amplifiers, broadband matching networks

## Abstract

This paper presents a strategy to design ultrawideband power amplifiers with a fractional bandwidth of approximately 200%. It exploits a simple output matching network, which consists of a series transmission line together with a shunt stub, to compensate the output parasitic network of the device. Following this, a multisection transformer is implemented to obtain the optimal load at the intrinsic drain plane. As design examples, several output matching networks were designed for two different size GaN HEMT devices. One of these examples was implemented and characterized, and a drain efficiency from 52% to 70% and an output power between 40 dBm and 42.5 dBm were obtained, over 67% of the 5G sub-6-GHz band (i.e., 0.1 to 4 GHz). The aforementioned results, to the best of the authors’ knowledge, represent the state of the art in broadband power amplifiers.

## 1. Introduction

Nowadays, 5G sub-6-GHz systems demand very high-speed transmission rates in order to be able to deliver the always increasing amount of data that are generated by millions of users around the world. This means that transmitters must cover large ranges of frequencies, and so they make use of a power amplifier (PA), which is responsible for increasing the wave energy with minimum waste, after the modulation process. Therefore, solutions to design high efficiency and wideband PAs are a challenge that is always present in the deployment of new communication networks.

In the literature, several approaches found hybrid PAs with a good efficiency for bandwidths wider than an octave. For instance, in [1], a high efficiency PA with 145.5% fractional bandwidth was obtained using a simplified device model. A 153.2% fractional bandwidth PA was obtained in [2], where an estimation of the power and efficiency contours was carried out in order to visualize a broadband output matching network (OMN). The design process shown in [3] used an optimization algorithm to design a broadband PA with equalized gain over the band, and a 145.6% fractional bandwidth was obtained. In [4], a design process based on L-sections was proposed, obtaining a high efficiency over a 152.9% fractional bandwidth. In addition, other methodologies have been implemented such as the real frequency technique [5,6,7], wideband class J [8], and class E [9], among other harmonic tuned PAs.

This paper presents an approach to design ultrawideband power amplifiers using a simple OMN, focused only on the fundamental optimal load. This involves the use of a series transmission line, a shunt stub, and a multisection transformer. Two examples, using two different GaN HEMT devices, are theoretically studied, and one of them is implemented and characterized as a demonstration circuit. The characterization of the implemented amplifier shows state-of-the-art results in terms of high-efficiency bandwidth. Its bandwidth extends from very low frequencies (i.e., 0.1 GHz) to 4 GHz, which means that the PA covers 67% of the 5G sub-6-GHz band. The obtained drain efficiency is between 52 and 70.7% over the band. A comparison with other state-of-the-art works is presented in Table 1.

## 2. Output Network Topology and Bandwidth Estimation

In [10,11], the effective use of an equivalent output reactive network was used to estimate the load dispersion, with respect to frequency, in high frequency field effect transistors (FET). This simplified method proposes a network formed by a shunt capacitor $C_{\mathrm{OUT}}$ and a series inductor $L_{\mathrm{OUT}}$, which separates the drain current generator and the drain pin plane. Usually, these two reactive elements are referred to as the output parasitics or the parasitic output network of the device. They are an unavoidable characteristic of the device, which must be considered as part of the output matching network (OMN) or as something undesirable to be compensated for.

**Figure 1:**
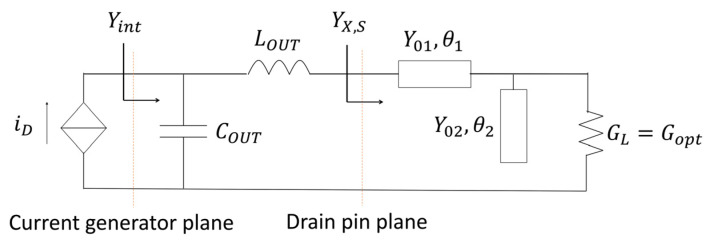
Proposed OMN.

Based on the above, this was the start point in this work. Whether the parasitic output network is well compensated for a large bandwidth, is an important concept to exploit, as this bandwidth can in theory be achieved, because with a wideband, real to real matching is possible through a multi-section transformer. Let us consider a reference frequency $f_{r}$, which is used for all of the calculations presented in the following equations. It can be demonstrated that a simple network involving a series transmission line and an open shunt sufficiently compensate the parasitic effects through the correct selection of their electrical lengths and characteristic admittances. Moreover, the bandwidth will be directly related to $f_{r}$. Thus, the proposed solution is shown in Figure 1, where $i_{D}$ is the drain current, and the elements $C_{\mathrm{OUT}}$ and $L_{\mathrm{OUT}}$ represent the estimated output parasitic network of the device.

Therefore, the design consists of finding the appropriate values for the transmission lines’ characteristic admittances and electrical lengths. As the function of the distributed network is to compensate $\;C_{\mathrm{OUT}}$ and $L_{\mathrm{OUT}}$, the value of the real load $G_{L}$ is chosen as $G_{\mathrm{opt}}$, where $G_{\mathrm{opt}}$ is the optimal load for tuned load conditions in order to simultaneously obtain the voltage and current saturation (i.e., $I_{\mathrm{MAX}}/\left[2\left(V_{\mathrm{DD}}-V_{k}\right)\right]$). Looking at Figure 1, the external load admittance at the drain pin is given by

(1) $$Y_{X,S}=Y_{01}\frac{G_{L}+j\left(Y_{01}T_{1}+T_{2}\right)}{Y_{01}-T_{1}T_{2}+{\mathrm{jG}}_{L}T_{1}}$$

where $T_{1}=\tan\theta_{1}$ and $T_{2}=Y_{02}\tan\theta_{2}$.

However, the optimal external load to perfectly compensate for the parasitic effects $C_{\mathrm{OUT}}$ and $L_{\mathrm{OUT}}$, $Y_{X,O}$ must be equal to

(2) $$Y_{X,O}=\frac{G_{\mathrm{opt}}-{j\omega C}_{\mathrm{OUT}}}{1-\omega^{2}L_{\mathrm{OUT}}C_{\mathrm{OUT}}-{j\omega L}_{\mathrm{OUT}}G_{\mathrm{opt}}}=G_{X,O}+{\mathrm{jB}}_{X,O}$$

Thus, the solution for the reference frequency $f_{r}$ is obtained by equalizing (1) and (2), which leads to the following pair of equations,

(3) $$G_{X,O}T_{1}T_{2}+B_{X,O}G_{L}T_{1}=Y_{01}G_{X,O}-Y_{01}G_{L}$$

(4) $$[Y_{01}^{2}-G_{X,O}G_{L}]T_{1}+Y_{01}T_{2}+B_{X,O}T_{1}T_{2}=Y_{01}B_{X,O}$$

Solving (3) and (4) for $T_{1}$, a quadratic equation is obtained,

(5) $$\left[G_{X,O}\left(Y_{01}^{2}-G_{X,O}G_{L}\right)-B_{X,O}^{2}G_{L}\right]T_{1}^{2}-2Y_{01}B_{X,O}G_{L}T_{1}+Y_{01}^{2}\left(G_{X,O}-G_{L}\right)=0$$

Therefore, $T_{1}$ is given by

(6) $$T_{1}=\frac{Y_{01}B_{X,O}G_{L}\pm \sqrt{Y_{01}^{2}B_{X,O}^{2}G_{L}^{2}-Y_{01}^{2}\left[G_{X,O}\left(Y_{01}^{2}-G_{X,O}G_{L}\right)-B_{X,O}^{2}G_{L}\right]\left(G_{X,O}-G_{L}\right)}}{G_{X,O}\left(Y_{01}^{2}-G_{X,O}G_{L}\right)-B_{X,O}^{2}G_{L}}$$

The sign before the square root is chosen to obtain a positive value for $T_{1}$, and the electrical length $\theta_{1}$ is obtained as

(7) $$\theta_{1}=\arctan T_{1}$$

As shown in Figure 1, $G_{L}=G_{\mathrm{opt}}$ is assumed to guarantee the optimal load, even at very low frequencies. Now, using (3),

(8) $$T_{2}=\frac{Y_{01}\left(G_{X,O}-G_{L}\right)-B_{X,O}G_{L}T_{1}}{G_{X,O}T_{1}}$$

and the electrical length $\theta_{2}$ is given by

(9) $$\theta_{2}=\arctan\left(\frac{T_{2}}{Y_{02}}\right)$$

Notice that (7) and (9) depend on the characteristic admittances $Y_{01}$ and $Y_{02}$. In this framework, they are left as the free selection variables. However, an estimation of the bandwidth is recommended to evaluate the selection of these variables. To this purpose, as a criterion for estimating the bandwidth, the following intrinsic reflection coefficient is defined as

(10) $$\Gamma_{\mathrm{int}}=-\frac{Y_{\mathrm{int}}-G_{\mathrm{opt}}}{Y_{\mathrm{int}}+G_{\mathrm{opt}}}$$

with

(11) $$Y_{\mathrm{int}}={j\omega C}_{\mathrm{OUT}}+\frac{Y_{X,S}\left(\frac{1}{{j\omega L}_{\mathrm{OUT}}}\right)}{Y_{X,S}+\frac{1}{{j\omega L}_{\mathrm{OUT}}}}$$

Thus, the bandwidth here is defined by $\Gamma_{\max}$, the maximum value of $\left|\Gamma_{\mathrm{int}}\right|$ that can be tolerated.

## 3. Design and Implementation

Let us consider two different devices to demonstrate the effectiveness of the design equations proposed in Section 2. These are the Wolfspeed (Durham, NC, USA) CG2H40010 and CG2H40025 GaN HEMT devices. Table 2 shows the values for $C_{\mathrm{OUT}}$ and $L_{\mathrm{OUT}}$ for both devices, respectively.

**Figure 2:**
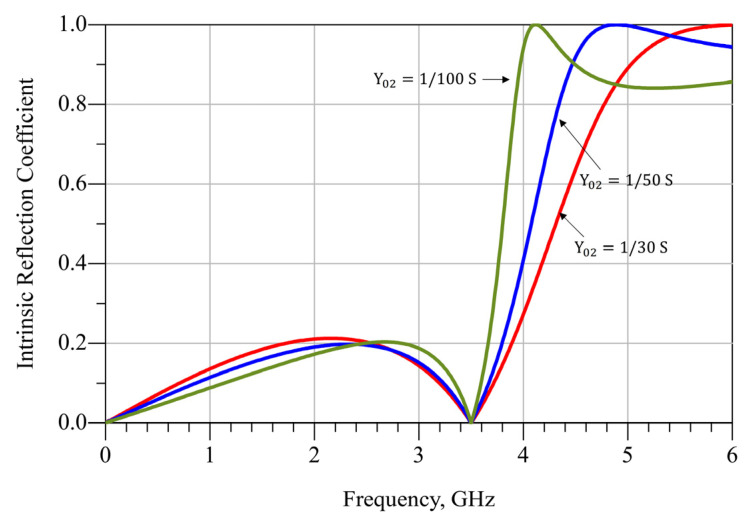
$\left|\Gamma_{\mathrm{int}}\right|$ for different values of $Y_{02}$ using the CG2H40010 device.

In this case, $Y_{01}$ is selected in order to match the physical dimensions of the device’s drain pin. Both devices have a drain pin width of 1.4 mm. Hence, $Y_{01}=\frac{1}{46}\;S$ is a good selection for a Taconic RF35 substrate with $\epsilon_{r}=3.5\;$ and 0.76 mm in height. This value ensures the needed soldering space at the drain pin. $Y_{02}$ is chosen only looking at $\Gamma_{\mathrm{int}}$, as given in Equation (10). As an example, let us consider the CG2H40010 device and $f_{r}=3.5\;\mathrm{GHz}$. Using (6)–(9), for different implementable values of $Y_{02}$, $\theta_{1}$ and $\theta_{2}$ are obtained, as presented in Table 3. Figure 2 shows $\left|\Gamma_{\mathrm{int}}\right|$ versus frequency, which provides a notion of the expected bandwidth. It is noticeable that, if $\Gamma_{\max}=0.2$ is considered, a bandwidth from almost 0 Hz to 4 GHz is expected for $Y_{02}=\frac{1}{30}\;S$. As can be seen in Figure 2, for greater values of $Y_{02}$, the bandwidth tends to increase slightly. Therefore, $Y_{02}=\frac{1}{30}\;S$ is considered to be a very good implementable selection in this work.

**Figure 3:**
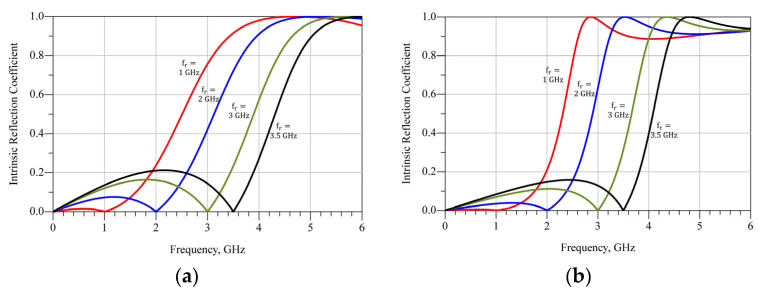
$\left|\Gamma_{\mathrm{int}}\right|$ for different values of $f_{r}$, (**a**) using CG2H40010 and (**b**) CG2H40025.

With the values of $Y_{01}$ and $Y_{02}$, using (6)–(11), $\left|\Gamma_{\mathrm{int}}\right|$ can also be studied for different values of $f_{r}$. This is shown in Figure 3 for both devices. In Table 4, the obtained values of $\theta_{1}$ and $\theta_{2}$ versus $f_{r}$ are presented.

**Figure 4:**
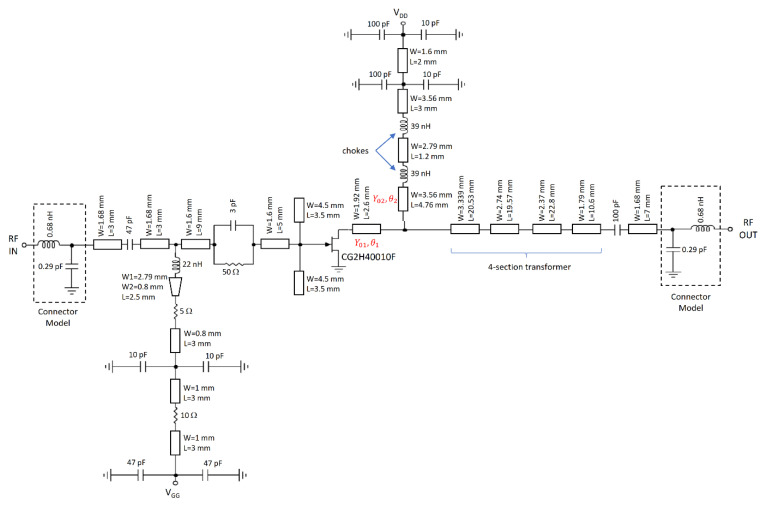
Schematic of the designed PA.

**Figure 5:**
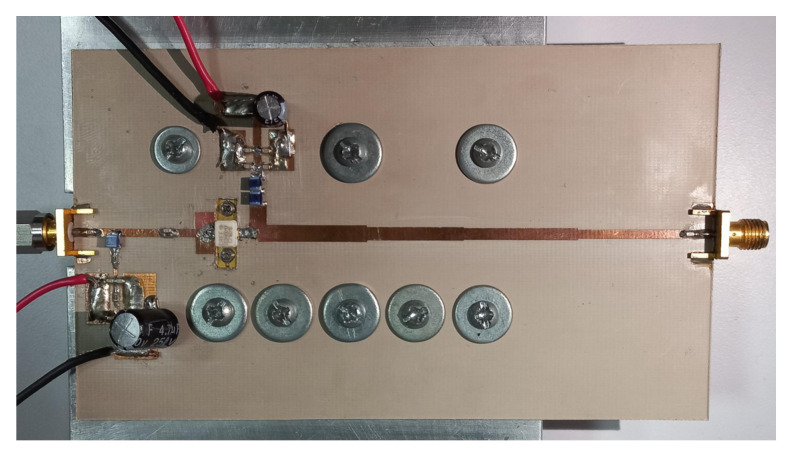
Picture of the designed PA. Size: 12.8 cm × 5 cm.

As the interest of this work is to design an amplifier that covers most of the 5G sub-6-GHz band, $f_{r}=3.5\;\mathrm{GHz}$ together with CG2H40010 were selected as a suitable option for implementation and case study. However, the complete PA design can also be carried out using CG2H40025, as demonstrated in [2], where an Input Matching Network (IMN) and stability network were designed for broadband applications for this device. Thus, the PA schematic is shown in Figure 4. As mentioned above, the substrate RF 35 with $\epsilon_{r}=3.5$ and h=0.76 mm was used. As usual, the IMN has been designed including two low impedance shunt stubs and an RC stability network. Two choke inductors in series were implemented as part of the bias-T at the OMN, and the typical 50-ohm terminal load was transformed to $G_{L}=G_{\mathrm{opt}}=\frac{1}{25}\;S$ through a four-section Chebyshev transformer. A class AB bias point was adopted with $V_{\mathrm{DD}}=28\;V$ and $V_{\mathrm{GG}}=-2.7\;V$ ($I_{\mathrm{DD}}=150\;\mathrm{mA}$). The implemented PA is presented in Figure 5. In this case, an ad hoc aluminum carrier was used as a heat sink.

**Figure 6:**
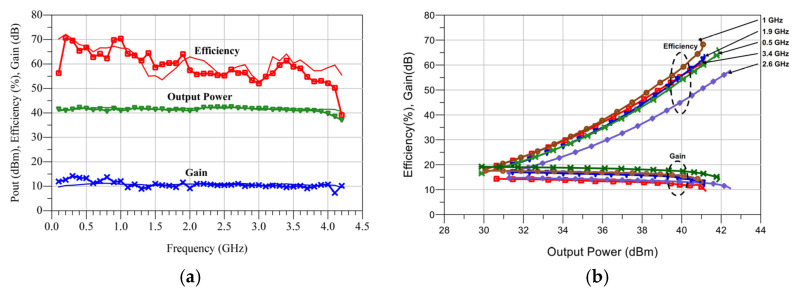
(**a**) CW characterization versus frequency. Solid lines: simulation results. Symbols: measurements. (**b**) Measured output power and efficiency profiles.

The Continuos Wave (CW) characterization results are shown in Figure 6. As can be noticed, a 4 GHz bandwidth was obtained from very low frequencies up to 4 GHz. Over this band, a drain efficiency between 52% and 70% was obtained, while the output power was from 40 dBm to 42.5 dBm. The transducer gain was always higher than 9 dB over the band. To the best of the authors´ knowledge, these results represent a state-of-the-art single stage PA design.

## Figures and Tables

**Table 1:** Comparison of the designed and implemented PA with the state-of-the-art.

Ref.	BW (GHz)	Gain (dB)	Output Power (dBm)	PAE (%)	Drain Efficiency (%)
[1]	0.6–3.8	9–14	40–41.9	46–75	51–76
[2]	0.45–3.4	8–10.5	41.5–44.3	54–70.4	-
[3]	0.85–5.4	8–9.5	43.5–44.9	45–55	-
[4]	0.4–3	10–12	40–42.5	53–72	-
This work	0.1–4	9–14	40–42.5	48–68	52–70.7

**Table 2:** Parasitic estimations for the CG2H40010 and CG2H40025 GaN HEMT devices.

Device	$L_{OUT}$	$C_{OUT}$	$G_{opt}$
CG2H40010	0.45 nH	2.03 pF	1/25 S
CG2H40025	0.49 nH	3.39 pF	1/14 S

**Table 3:** $\theta_{1}$ and $\theta_{2}$ calculations using Equations (6)–(9) for CG2H40010 with $f_{r}=3.5\;\mathrm{GHz}$.

$Y_{01}$	$Y_{02}$	$\theta_{1}$	$\theta_{2}$
1/46 S	1/30 S	19.5°	51.3°
1/46 S	1/50 S	19.5°	64.4°
1/46 S	1/100 S	19.5°	76.5°

**Table 4:** $\theta_{2}$ calculations for CG2H40010 and CG2H40025 for different values of $f_{r}$.

	$f_{r}=1\;GHz$		$f_{r}=2\;GHz$		$f_{r}=3\;GHz$		$f_{r}=3.5\;GHz$	
Device	$\theta_{1}$	$\theta_{2}$	$\theta_{1}$	$\theta_{2}$	$\theta_{1}$	$\theta_{2}$	$\theta_{1}$	$\theta_{2}$
CG2H40010	18.1°	19.2°	23.9°	35.1°	21.9°	46.8°	19.5°	51.3°
CG2H40025	6°	31.5°	7.9°	51°	5.9°	61.9°	4°	65.6°
